# Effects of Drugs of Abuse on the Blood-Brain Barrier: A Brief Overview

**DOI:** 10.3389/fnins.2020.00513

**Published:** 2020-05-21

**Authors:** Emely Pimentel, Kalaiselvi Sivalingam, Mayur Doke, Thangavel Samikkannu

**Affiliations:** ^1^School of Medicine, St. George’s University, Great River, NY, United States; ^2^Department of Pharmaceutical Sciences, Irma Lerma Rangel College of Pharmacy, Texas A&M University, Kingsville, TX, United States

**Keywords:** blood-brain barrier, cocaine, methamphetamine, morphine, heroin, nicotine

## Abstract

The use of psychostimulants and alcohol disrupts blood-brain barrier (BBB) integrity, resulting in alterations to cellular function, and contributes to neurotoxicity. The BBB is the critical boundary of the central nervous system (CNS) where it maintains intracellular homeostasis and facilitates communication with the peripheral circulation. The BBB is regulated by tight junction (TJ) proteins that closely interact with endothelial cells (EC). The complex TJ protein network consists of transmembrane proteins, including claudins, occludins, and junction adhesion molecules (JAM), as well as cytoskeleton connected scaffolding proteins, zonula occludentes (ZO-1, 2, and 3). The use of psychostimulants and alcohol is known to affect the CNS and is implicated in various neurological disorders through neurotoxicity that partly results from increased BBB permeability. The present mini review primarily focuses on BBB structure and permeability. Moreover, we assess TJ protein and cytoskeletal changes induced by cocaine, methamphetamine, morphine, heroin, nicotine, and alcohol. These changes promote glial activation, enzyme potentiation, and BBB remodeling, which affect neuroinflammatory pathways. Although the effect of drugs of abuse on BBB integrity and the underlying mechanisms are well studied, the present review enhances the understanding of the underlying mechanisms through which substance abuse disorders cause BBB dysfunction.

## Introduction

The blood-brain barrier (BBB) is formed by an endothelial cell (EC) monolayer between the blood and central nervous system (CNS) that contributes to maintaining structural and functional homeostasis in the brain. The BBB structure interacts with perivascular pericytes, microglial cells, astrocytes, and neurons that, together, form the neurovascular units (Abbott et al., 2010; Obermeier et al., 2013; Chow and Gu, 2015; Banks, 2016). Notably, BBB permeability is –in part- a function of pericyte-regulated endothelial transcytosis. The BBB is formed by an EC network rigidly connected by complex junction systems comprised of smaller trans-membrane tight junction (TJ) proteins, including junction adhesion molecules (JAM), endothelial cell-selective adhesion molecules, occludins, and claudins (Ballabh et al., 2004; Van Itallie and Anderson, 2014). This creates a boundary between the CNS and peripheral circulation for regulating blood-CNS exchange (Kousik et al., 2012).

The BBB is critical to the maintenance of brain homeostasis as it regulates the entry of macromolecules, ions, and neurotransmitters from the blood to the brain (Abbott et al., 2010; Lippmann et al., 2013; Wilhelm and Krizbai, 2014; Erickson and Banks, 2018). Notably, the BBB limits the entry of neurotoxic substances from the periphery and contributes to maintenance of a stable microenvironment for optimal neuronal function to prevent critical CNS damage (Abbott et al., 2010). This highly selective permeable barrier allows passive diffusion of certain gases, water, and lipid-soluble molecules, which is necessary for efficient neural function (Bellettato and Scarpa, 2018). Recent research has found that drugs of abuse, including cocaine, methamphetamine (METH), morphine, heroin, nicotine, and alcohol, cause BBB dysfunction by altering TJ formation and protein expression (Hawkins and Davis, 2005; Abbott et al., 2006). The concentration and distribution of a drug regulate its passage (Pardridge, 2012).

Globally, studies have shown that approximately 240 million people are alcohol-dependent, more than one billion are smokers, and roughly 15 million are illicit drug users. Substance use disorder is either directly or indirectly responsible for 11.8 million annual deaths; moreover, the use of different drugs varies across geographical locations. In 2017, 70,237 people in the USA died from drug overdoses (Kariisa et al., 2019). The National Survey on Drug Use and Health estimated that approximately 20 million Americans have used illicit drugs within the past month, which is expected to reach 9.2% of the United States population. Moreover, there are significant gender-based differences in the initiation of drug usage, as well as neurotransmitter systems and neural circuitries, among individuals with substance use disorders. Individual differences in addiction behaviors depend on several factors, including the method of drug administration, sociocultural factors, genetics, personality traits, and several biological processes (Becker et al., 2017). Furthermore, preclinical studies have shown that females often show higher responsivity to drugs compared to males.

The menstrual cycle and estrogen are essential for treatment outcome in female drug users. Specifically, withdrawal symptom severity may differ between the luteal and follicular menstrual phase (Snively et al., 2000; Terner and de Wit, 2006; Allen et al., 2010). Males have a higher metabolic rate, which affects neural mechanisms (Fattore et al., 2014). Regardless, both males and females exhibit brain changes after using drugs of abuse (Leyton, 2007; Wegener and Koch, 2009; Willuhn et al., 2010; Andersen et al., 2012).

**FIGURE 1:**
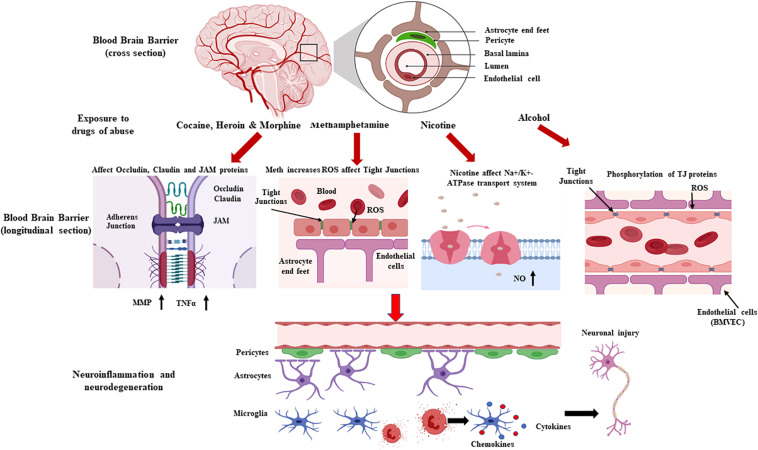
Schematic representation showing drug-induced loss of blood brain barrier (BBB) permeability and the associated neurodegeneration. The neurovascular unit and the BBB are affected by various drugs of abuse, which alter vessel permeability via disruption of tight junction proteins complexes (junction adhesion molecules, endothelial cell-selective adhesion molecules, occludins, and claudins), transport systems, and intracellular signaling. BBB disruption, which affects immune cell transmigration and neuroinflammation and contributes to an imbalanced redox system, affects the brain’s microenvironment and homeostasis, leading to neurotoxicity (Created with Biorender.com).

Drugs of abuse increase BBB permeability, which, in turn, increases influx of peripheral toxins into the brain. Consequently, BBB dysfunction activates neuro-inflammatory pathways by increasing astro-glial activation, which –in turn- increases BBB permeability and susceptibility of the CNS to foreign molecules (Kousik et al., 2012; Ronaldson and Davis, 2015). BBB integrity loss contributes to changes in transport pathways, disruption of EC-cell interactions, redistribution, and/or downregulation of TJ protein modifications (Kousik et al., 2012; Rochfort et al., 2014; Yang et al., 2019). The present review summarizes the signaling mechanisms that contribute to drug abuse-related BBB dysfunction (Figure 1).

## Cocaine

The World Drug Report estimates that worldwide, 18.8 million people used cocaine in 2014 (United Nations Office on Drugs and Crime, 2016). In 2016, the National Institute on Drug Abuse reported an age-adjusted cocaine-mediated death rate of 52.4% in the USA. Cocaine is a highly addictive stimulant that restricts dopamine and monoamine reuptake through dopamine transporter (DAT) antagonism (Kousik et al., 2012). Monoamine oxidase inhibition leads to imbalanced free-radical production, which generates oxidative stress and neuroinflammation. Continuous cocaine administration has been shown to contribute to a 50% increase in BBB permeability, with a concomitant decrease in trans endothelial electrical resistance (TEER) due to basement membrane and neurovascular capillary disruption, due to up-regulated matrix metalloproteinase (MMP) and tumor necrosis factor (TNF-α) expression (Sharma et al., 2009). Moreover, TJ protein loss and alteration, specifically decreased JAM-2 and zonula occludens-1 (ZO-1) levels, are characteristic of cocaine transit across the BBB (Dietrich, 2009). CCL2 (C-C motif chemokine ligand-2) and CCR2 (C-C motif chemokine receptor-2) expression upregulation has also been reported (Fiala et al., 2005). Cocaine use affects intercellular junctions and causes cell ruffling, which contributes to increased permeability and decreased TEER values across BBB monolayers (Fiala et al., 2005; Srinivasan et al., 2015).

An alternate pathway for cocaine-induced BBB permeability alteration involves platelet-derived growth factor (PDGF) intermediates (Yao et al., 2011). Cocaine binding to sigma receptors evokes a proteolytic signal cascade that initiates PDGF-B chain assembly, a fundamental intermediate for increased membrane permeability that inhibits store-operated calcium entry (Yao et al., 2011; Cristina Brailoiu et al., 2016; Rosado, 2016). Moreover, cocaine binding to sigma receptors has been associated with dopamine uptake inhibition and enhanced dopamine release that neutralizes the effects of antibody reversal on increased PDGF expression (Kumar, 2011). In rats, chronic cocaine exposure has been shown to increase BBB permeability in the hippocampus and striatum, suggesting that the hippocampus could be affected by glial and cytokine migration without significant changes in cortical or cerebellar permeability (Riley et al., 2017). Furthermore, it has been recently revealed that acute cocaine administration alters BBB permeability and may increase neurotoxicity in free-moving rats (Barr et al., 2019).

Astrocytes have complex morphologies involving extensive processes that communicate within the neurovascular unit and maintain the BBB. Cocaine exposure potentiates aberrant astroglial responses in cellular and animal models, which leads to loss of BBB integrity and function (Fattore et al., 2002; Yang et al., 2016). Other studies have reported cocaine-induced neuroinflammation and BBB disruption mediated by the activation of brain microglial cells to secrete several cytokines, chemokines, and other neurotoxic factors (Yao et al., 2010; Buch et al., 2012). Cocaine upregulates these inflammatory mediators and cell adhesion molecules, including intercellular adhesion molecule-1, vascular cell adhesion molecule, and activated leukocyte cell adhesion molecule in the BBB endothelium (Fiala et al., 1998; Yao et al., 2011).

Previous *in vitro* findings have shown that exposure of pericytes to cocaine upregulates pro-inflammatory cytokines [TNF-α, interleukin (IL)-1β, and IL-6] in both intracellular and extracellular compartments. In addition, cocaine activates the Src–PDGFR-β–NF-κB pathway, which enhances CXCL10 [chemokine (C-X-C motif) ligand-1] secretion. This causes increased neuroinflammation in human brain vascular pericytes (Table 1), which further leads to neurovascular unit disruption and immune cell transmigration across the BBB (Niu et al., 2019; Sil et al., 2019).

**TABLE 1:** Summary of cocaine- and methamphetamine-induced neurotoxicity based on their effect on the structural integrity of the blood brain barrier and their respective molecular pathways.

Drugs	Pathways involving in BBB leakiness-gene/proteins of interest	Effect on BBB
Cocaine	Activated leukocyte cell adhesion molecule (ALCAM)	↑
	C-C Motif CheniokineLigand-2 (CCL2)	↑
	C-C Motif Chemokine receptor- 2 (CCR2)	↑
	CXCL10(eheniokine (C-X-C motif) ligaud-1)	↑
	Intercellular adhesion molecule-1 (ICAM-1)	↑
	Junction n 1 adhesion molecule 2 (JAM-2)	↓
	Matrix metalloproteinase (MMP)	↑
	Pro-inflammatory cytokines (TNF-α, IL-l β, and IL-6)	↑
	Src-PDGFR-p-NF-KB	↑
	Tumor Necrosis factor-alpha (TNF-α)	↑
	Vascular cell adhesion molecule (VCAM),	↑
	Zonula occludens-1 (ZO-1)	↓
METH	Actin related protein 2/3 (3Arp2/3) complex	↑
	Claudin-5	↓
	Glial fibrillary acidic protein (GFAP)	↑
	Glucose transporter (GLUT1)	↓
	Glutathione (GSH)	↓
	Interleukin (IL)-6	↑
	Interleukin (IL)-8	↑
	Matrix metalloproteinase- 9 (MMP-9)	↑
	Matrix metalloproteinase-1 (MMP-1)	↑
	Nuclear transcription factor- KB (NF-KB)	↑
	p53 upregulated modulator of apoptosis (PUMA)	↑
	Reactive oxygen species (ROS)	↑
	Rho-associated protein kinase (RhoA ROCK)	↑
	Sigma-1 receptor	↑
	Tumor Necrosis factor-alpha (TNF- α)	↑
	Zonula occludens-1 (ZO-1)	↓

## METH

METH is a highly addictive and illicit psychostimulant and is the second most widely abused drug in the USA. It adversely affects brain homeostasis through BBB dysfunction and hyperthermia (O’Shea et al., 2014). Its high lipophilicity allows for rapid and comprehensive transmigration across the BBB. METH binding to the DAT induces reversal transport of norepinephrine, serotonin (5HT), and dopamine, which causes their excessive release into the synapse (Kousik et al., 2012). Moreover, it inhibits monoamine reuptake that leads to post-synaptic cleft stimulation (Kousik et al., 2012). Chronic METH administration causes irreversible impairment of serotonin and dopamine transport into synaptic terminals in various brain regions, especially in the hippocampus.

Various METH dosing paradigms significantly disturb endothelial TJ assembly by inducing downregulation, fragmentation, or redistribution of major TJ proteins, including claudin-5 and ZO-1, which are mediated by MMP-1 and MMP-9 peptidases. This leads to reduced endothelial barrier tightness and increased BBB paracellular permeability (Mahajan et al., 2008; Ramirez et al., 2009; Banerjee et al., 2010; Liu et al., 2012; Toborek et al., 2013; Sajja et al., 2016; Rubio-Araiz et al., 2017). Moreover, repeated intravenous METH administration downregulates TJ proteins, which causes glutathione depletion and increases endothelial reactive oxygen species (ROS) levels. This triggers actin polymerization that possibly involves activation of actin-related protein 2/3 complex or myosin light chain kinase and its downstream target RhoA (Mahajan et al., 2008; Ramirez et al., 2009; Banerjee et al., 2010; Park et al., 2013). In mice, research has shown that METH-induced glucose transporter and uptake downregulation is an important causative factor for BBB integrity loss (Abdul Muneer et al., 2011). Further, METH reduces TJ protein expression, rearranges the F-actin cytoskeleton, and increases BBB permeability through Rho-associated protein kinase-dependent pathway activation in the frontal lobes and isolated primary microvascular endothelial cells (Xue et al., 2019).

Other neurotoxicity mechanisms have also been suggested, including the METH-induced increase in reactive oxidative stress and ROS levels, which activate myosin light chain protein kinase, thereby reducing TJ protein expression (Gonçalves et al., 2010). Additionally, METH-induced TJ protein downregulation and resulting BBB integrity disruption may involve activation of NF-κB transcription and pro-inflammatory cytokines (TNF-α) in BBB endothelial cells (Coelho-Santos et al., 2015; Rom et al., 2015). METH transit across the BBB damages the nucleus accumbens shell region and prefrontal cortex and causes hyperthermia, neuroinflammation, and brain edema (Kousik et al., 2012). Recent studies have reported METH-induced pericyte migration via sigma-1 receptor activation, p53 upregulated modulator of apoptosis expression, and downstream mitogen-activated protein kinase and Akt/PI3K pathways in C3H/10T1/2 cells, leading to BBB dysfunction (Zhang et al., 2017). METH-activated microglia and astrocytes in the neurovascular unit may promote neurotoxicity and astroglial reactivity and induces BBB integrity loss (Asanuma et al., 2004; Dietrich, 2009). In addition, METH increases the expression of the glial fibrillary acidic protein, σ1 receptors, TNF-α, IL-6, and IL-8 in mouse and rat astrocytes. This leads to METH-induced inflammation in microglial cells where increased TNF-α release can activate BBB endothelium, which increases transmigration of circulating leukocytes through the leaky BBB (Malaplate-Armand et al., 2005; Shah et al., 2012; Zhang et al., 2015; Table 1).

## Morphine

Opioids are widely-used analgesics that bind with opioid and/or toll-like receptors (TLR) in the CNS (Chaves et al., 2017; Yang et al., 2018). Transcellular solute and xenobiotic transport across the BBB is selectively controlled by the local influx and efflux transporters, including ATP-binding cassette (ABC), P-glycoprotein (P-gp, ABCB1), breast cancer resistance protein (ABCG2), multidrug resistance-associated proteins (ABCC) transporters, and solute carrier transporters (Abbott et al., 2010; Chaves et al., 2017). Among the four central opioid receptor families [mu (μ), delta (δ), kappa (κ), and opioid receptor like-1 (ORL1) receptor], μ-opioid receptors are primarily responsible for the analgesic effects. Microvascular endothelial cells have high affinity and specific opiate binding sites that mediate morphine’s effects on the CNS (Stefano et al., 1995).

Morphine exerts its effects by directly acting on the CNS with its illicit use leading to tolerance and drug dependence (Gach et al., 2011). Drug transmigration is essential to psychological dependence. Morphine alters BBB homeostasis and permeability through pro-inflammatory cytokine activity, intracellular calcium release dysregulation, and myosin light chain protein kinase activation, which results in ROS-mediated neurotoxicity (Kousik et al., 2012).

P-gp limits the net transport of several foreign substrates into the brain through active unidirectional efflux. This transporter regulates foreign-agent pharmacokinetics in the brain by inhibiting or augmenting their movement across the BBB, which restrains morphine entry into the brain (Tournier et al., 2011). Moreover, P-gp attenuates morphine-induced migratory properties and transcriptional effects (Miller, 2010). Acute morphine treatment inhibits P-gp expression, which increases morphine uptake in the brain, which modifies the acute analgesic and locomotive morphine effects and selectively alters critical transcriptional responses in the nucleus accumbens (Seleman et al., 2014). This indicates that the transporter system significantly contributes to mediating BBB integrity and permeability of carrier mediated transport (Table 2).

**TABLE 2:** Summary of morphine-, heroin-, nicotine-, and alcohol-induced neurotoxicity according to their effect on the structural integrity of the BBB and their respective molecular pathways.

Drugs	Pathways involving in BBB leakiness-gene/proteins of interest	Effect on BBB
Morphine	Myosin light chain protein kinases	↑
	P-glycoprotein	↓
	Reactive oxygen species (ROS)	↑
Heroin	Junctional adhesion molecule-2 (JAM-2)	↑
	P-glycoprotein	↓
	Zonula occludens-1 (ZO-1)	↓
Nicotine	Claudin-1 and -5	N.D.
	Claudin-3	↓
	Junctional adhesion niolecule-l (JAM-1)	↓
	Nitric oxide (NO)	↑
	P-glycoprotein	↓
	Reactive oxygen species (ROS)	↑
	Zonula occludens-1 (ZO-1)	↓
	Zonula occludens-2 (ZO-2)	N.D.
Alcohol	ERK1/2 and p-38	↑
	Matrix metalloproteinase- 9 (MMP-9)	↑
	Matrix metalloproteinase-3 (MMP-3)	↑
	Reactive oxygen species (ROS)	↑
	Toll like receptor- 2 (TLR-2)	↑
	Toll like receptor-3 (TLR-3)	↑
	Toll like receptor- 4 (TLR-4)	↑
	Transient receptor potential cation channel (TRP7)	↓

## Heroin

There has been a rapid increase in opioid abuse in the USA with approximately 580 new heroin users every day. Deaths resulting from opiate overdose, including pain relievers and heroin, increased by 200% between 2000 and 2014 (Rudd et al., 2016). Heroin can be reversibly metabolized into morphine; upon selective transmigration across the BBB, heroin is transformed into morphine and metabolized into 6-monoacetylmorphine (6-MAM). The superior heroin lipophilicity allows faster transit across the BBB than morphine (Boix et al., 2013). The acetylation of both hydroxyl groups while synthesizing heroin increases its BBB penetration rate by 100-fold, which could contribute to its high addictive potential (Boix et al., 2013). These addictive properties are regulated by the μ-opioid receptor (MOR), which mediates the rewarding effects of heroin. A recent study reported that 6-MAM has a higher affinity for μ-opioid receptor G-protein activation than morphine (Seleman et al., 2014).

Heroin’s effects indirectly involve its metabolites (morphine and 6-MAM) that act as substrates in P-gp membrane regulation. Upon heroin transition into the brain, it has a higher synthesized concentration than morphine. This suggests that the metabolite is the primary effector of the detrimental effects of heroin on the BBB. In the extracellular brain fluid, these metabolites bind and activate MORs, which regulates crucial neurological automatic processes (Boix et al., 2013). P-gp inhibition at the BBB acutely disrupts the BBB permeability and selectivity in the nucleus accumbens (Seleman et al., 2014). Moreover, increased levels of these metabolites in the brain downregulate TJ protein expression, especially ZO-1, which increases BBB permeability. Contrastingly, there have been reports of increased JAM-2 TJ protein expression (Seleman et al., 2014; Table 2).

## Nicotine

Nicotine is a stimulant that acts as a nicotinic acetylcholine receptor agonist. Its high lipophilicity allows for rapid (10–20 s after inhalation) transit across the BBB. Chronic exposure to nicotine disrupts TJ proteins and results in an ionic imbalance within the BBB microenvironment. Consequently, this causes ischemic hypoxia and exacerbates stroke-associated brain edema and neuronal injury (Paulson et al., 2006; Bradford et al., 2011). Nicotine exposure alters BBB permeability through TJ protein modulation. It does not affect ZO-1, 2; claudin-1, -3; or -5 TJ protein expression, however, it disrupts the distribution of claudin-3 and ZO-1 TJ proteins (Kousik et al., 2012). Moreover, nicotine-induced BBB impairment has been shown to involve decreased ZO-1 expression, which affects brain homeostasis (Hutamekalin et al., 2008). Similarly, static- or flow-based *in vitro* BBB model studies have reported tobacco-induced alterations in TJ protein expression and re-distribution, which increases intracellular ROS/RNS and the secretory profile of various pro-inflammatory markers (Hossain et al., 2011; Naik et al., 2014). This oxidative stress promotes atherosclerotic lesions and injures biliary epithelial cells (BECs) and TJ proteins via low-density lipoprotein oxidation enhanced by ROS activity (Kousik et al., 2012). Moreover, this results in increased transcytotic activity across the BBB through induced pinocytosis (Kousik et al., 2012).

Direct nicotine binding to nicotinic acetylcholine receptors on BECs induces acetylcholine-dependent nitric oxide (NO) release via activation of neurovascular endothelial NO synthase (Mazzone et al., 2010). Here, increased NO_2_ enhances vascular membrane permeability at the BBB. Furthermore, chronic nicotine administration compromises BBB integrity through TJ protein loss and alteration (ZO-1, claudin-3, and JAM-1). It affects regulated BBB transport and receptor systems that are essential for normal BBB function, as well as decrease the functional activity of ion transporters (Mazzone et al., 2010). Nicotine has been shown to decrease TEER and disturb the BBB transport system, which leads to increased xenobiotic uptake (Hutamekalin et al., 2008; Manda et al., 2010; Rodriguez-Gaztelumendi et al., 2011). Nicotine affects the functional activity of ion transporters, including Na^+^, K^+^, 2Cl^–^ cotransporter and Na^+^, K^+^-ATPase on BECs and inhibits P-gp activity in the CNS (Abbruscato et al., 2004; Paulson et al., 2006; Manda et al., 2010). Recent studies indicate that the H^+^/organic cation antiporter system is involved in blood-to-brain nicotine transport across BBB endothelial cells TR-BBB13 (Tega et al., 2018; Table 2). The precipitated ion gradient change induces brain edema, which further disrupts BBB integrity (Kousik et al., 2012).

## Alcohol

Alcohol is a widely used recreational drug responsible for 5.3% of deaths worldwide. In the USA, there are 23 million alcohol addicts with 88,000 people dying from alcohol use disorder. Alcohol acts on neurotransmitter receptors, including GABA, glutamate, and dopamine, with each receptor contributing to various physiologic effects, with chronic alcohol administration increasing tolerance and addiction (Burnett et al., 2016). Further, occasional alcohol consumption could lead to alcohol use disorder due to addiction and tolerance (Costin and Miles, 2014). Regular and excessive alcohol consumption causes brain injury, white matter loss, reduced brain volume, and neuronal loss associated with the BBB (Mann et al., 2001; Muneer et al., 2012; Bjork and Gilman, 2014). Moreover, gray matter loss is positively correlated with years of alcohol abuse (Fein et al., 2002). Chronic alcohol abuse induces neuroplastic changes and loss of neural circuit structure and strength (Mende, 2019).

The brains of individuals with alcohol dependence have increased proinflammatory cytokines, chemokines, microglial markers, and inflammasome proteins (He and Crews, 2008; Crews et al., 2013). Inflammatory cytokine and ROS activation contributes to BBB integrity disruption in TLR4-knockout mice (Rubio-Araiz et al., 2017). Further, postmortem alcoholic brains have shown increased TLR2, TLR3, and TLR4 expression in the orbitofrontal cortex, which correlates with BBB integrity loss (Crews et al., 2013). Moreover, they indicate that chronic alcohol intake increases TJ and neuroinflammatory protein (ERK1/2 and p-38) degradation, which may promote leukocyte brain infiltration (Rubio-Araiz et al., 2017).

Brain microvascular endothelial cells (BMVEC) are interconnected with TJ to form a tight monolayer in the BBB. Exposure of BMVEC to alcohol increases oxidative stress through myosin light chain and TJ protein phosphorylation. This leads to decreased TEER and increased leukocyte migration across the BBB (Haorah et al., 2007). Further, alcohol induces BBB dysfunction and neuroinflammation through MMP-3/9 activation and angiogenesis (VEGF)-A/VEGFR-2) impairment in primary endothelial cells in the brain (Muneer et al., 2012). Ethanol (EtOH) disrupts BBB integrity via endothelial transient receptor potential (TRP) channels, which affects the intracellular Ca^2+^ and Mg^2+^ dynamics. This increases endothelial permeability and alters inflammatory responses (Chang et al., 2018). EtOH-mediated TRPM7 expression downregulation causes BBB dysfunction and endothelium integrity loss (Macianskiene et al., 2008; Oh et al., 2012). Overall, TRP channels are involved in alcohol-mediated BBB dysfunction (Table 2).

## Conclusion

The BBB is crucial in drug abuse-mediated neurotoxicity. The BBB network characteristics are involved in functional restriction and transport control, as well as maintaining a constant CNS environment. TJ protein disruption, neuroinflammation, oxidative stress, and ROS production are fundamental mechanisms through which drugs alter the BBB structure and integrity. In adults, the mature CNS lacks substantial regenerative capacity while terminally differentiated neurons cannot divide and supplant themselves. Increased cell death due to neurotoxin entry could lead to a premature disabling condition. Although there has been previous research on the effects of drugs of abuse on the BBB, there is a need for further studies to identify novel therapeutic targets. Awareness regarding the effect of drugs of abuse on BBB integrity is paramount due to their toxic effects, which could induce immune reactions and neurodegeneration. There are current studies on potential therapeutic targets for preventing this neurotoxicity and propagation. Detailed knowledge regarding the physiology of drug abuse-associated BBB dysfunction, with respect to TJ protein complexes, transport systems, and intracellular signaling pathways, could allow the determination of effective therapeutic interventions. Moreover, a deep understanding of brain mechanisms could improve future prevention and treatment interventions. Comprehensive research on the mechanistic aspects of drug abuse-mediated BBB dysfunction could identify better therapeutic targets. Polysubstance abuse is among the significant challenges faced by drug abusers. Since each drug of abuse has a different mechanism of BBB disruption, understanding the effect of polysubstance abuse on BBB could allow the evaluation of novel therapeutic agents and systemic prediction of clinical efficacy. Future studies should explore means of restoring BBB integrity, which could extend scientific knowledge and contribute to novel therapeutic targets.

## Author Contributions

TS, EP, and KS designed and wrote the main manuscript. KS and MD contributed to the figure, reference sited, and proofread. All authors reviewed this manuscript.

## Conflict of Interest

The authors declare that the research was conducted in the absence of any commercial or financial relationships that could be construed as a potential conflict of interest.
